# Genome-wide association study for intramuscular fat deposition and composition in Nellore cattle

**DOI:** 10.1186/1471-2156-15-39

**Published:** 2014-03-25

**Authors:** Aline SM Cesar, Luciana CA Regitano, Gerson B Mourão, Rymer R Tullio, Dante PD Lanna, Renata T Nassu, Maurício A Mudado, Priscila SN Oliveira, Michele L do Nascimento, Amália S Chaves, Maurício M Alencar, Tad S Sonstegard, Dorian J Garrick, James M Reecy, Luiz L Coutinho

**Affiliations:** 1 Department of Animal Science, University of São Paulo, Piracicaba SP 13418-900, Brazil; 2 Embrapa Southeast-Cattle Research Center, São Carlos, SP 13560-970, Brazil; 3 Department of Genetics and Evolution, Federal University of São Carlos, São Carlos, SP 13565-905, Brazil; 4 United States Department of Agriculture, Agricultural Research Service, Bovine Functional Genomics Laboratory, Beltsville, Maryland 20705, USA; 5 Department of Animal Science, Iowa State University, Ames, IA 50011, USA

**Keywords:** Fatty acid, GWAS, *Bos indicus*, Beef, Positional candidate gene

## Abstract

**Background:**

Meat from *Bos taurus* and *Bos indicus* breeds are an important source of nutrients for humans and intramuscular fat (IMF) influences its flavor, nutritional value and impacts human health. Human consumption of fat that contains high levels of monounsaturated fatty acids (MUFA) can reduce the concentration of undesirable cholesterol (LDL) in circulating blood. Different feeding practices and genetic variation within and between breeds influences the amount of IMF and fatty acid (FA) composition in meat. However, it is difficult and costly to determine fatty acid composition, which has precluded beef cattle breeding programs from selecting for a healthier fatty acid profile. In this study, we employed a high-density single nucleotide polymorphism (SNP) chip to genotype 386 Nellore steers, a *Bos indicus* breed and, a Bayesian approach to identify genomic regions and putative candidate genes that could be involved with deposition and composition of IMF.

**Results:**

Twenty-three genomic regions (1-Mb SNP windows) associated with IMF deposition and FA composition that each explain ≥ 1% of the genetic variance were identified on chromosomes 2, 3, 6, 7, 8, 9, 10, 11, 12, 17, 26 and 27. Many of these regions were not previously detected in other breeds. The genes present in these regions were identified and some can help explain the genetic basis of deposition and composition of fat in cattle.

**Conclusions:**

The genomic regions and genes identified contribute to a better understanding of the genetic control of fatty acid deposition and can lead to DNA-based selection strategies to improve meat quality for human consumption.

## Background

Many consumers associate consumption of fat from beef with coronary heart disease, diabetes and obesity, due to the presence of cholesterol, high concentration of saturated fatty acids (SFA), and low concentration of polyunsaturated fatty acids (PUFA). However, consumption of fatty acids is necessary for human nutrition [1]. Beef has high nutritional value from children to seniors, is a rich source of protein (essential amino acids), iron, zinc, B vitamins and essential polyunsaturated fatty acids such as linoleic and linolenic acid [2]. Beef fat also has a high concentration of monounsaturated fatty acids (MUFA), whose melting point is low and can reduce the concentration of bad cholesterol (LDL) in blood circulation [3].

The amount of fatty acid and its composition in beef varies by breed, nutrition, sex, age and carcass finishing level [4]. The difficulties associated with determining intramuscular fat (IMF) deposition and composition as well as the limited knowledge on the genetic mechanisms that control these traits has limited genetic progress in the production of healthier beef.

The development of high-density bovine genotyping [5] and their use in genome-wide association studies (GWAS) have allowed identification of genomic regions associated with phenotypes of interest. The technique of GWAS exploits differences in allele frequencies of thousands of polymorphic markers available in unrelated individuals who possess different phenotypes (for example, deposition and composition of intramuscular fat), and leads to the identification of markers associated with a given phenotype [6]. Bayesian approaches have been applied to GWAS to detect significant quantitative trait loci (QTL) for traits of economic importance. One such approach uses multiple regression (evaluating marker effects simultaneously), treating marker effects as random to reduce overestimation bias of significant QTL effects, generating the actual posterior distribution of QTL effects given the data which can provide richer inference than can be obtained by simply constructing p-values as well as providing an alternative to the use of p-values to avoid false positives [7-9].

Brazilian beef is exported and consumed in more than 100 countries [10]. Purebred and crossbred Nellore cattle, which are of *Bos indicus* descent, are the predominant source of beef in Brazil. Previous research has documented that muscle and fat tissues from *Bos indicus* cattle develop in a different manner than in *Bos taurus* breeds [11-14]. However, studies documenting the genetics of fatty acid deposition and composition in *Bos indicus* breeds are limited.

In this study we performed a GWAS using high-density single nucleotide polymorphism (SNP) chips (770 k) and Bayesian methods (Bayes B) to identify genomic regions associated with fat deposition and fatty acid composition (FA) in Nellore beef.

## Results and discussion

### Intramuscular fat deposition and composition

Modern consumers are concerned with their overall health and often desire to reduce their caloric intake. This has increased the demand for lean meat production with a healthier fatty acid composition, which would comprise a lower proportion of SFA and greater proportion of MUFA. The amount of fat deposition in meat represented as IMF (mean = 2.77%) reported in this feedlot-finished study was greater than for pasture-finished counterparts, as expected, but lower than normally observed in Continental and English breeds [15-17]. Despite of that, IMF observed in this work was within limits of reasonable amount of fat to assure acceptable quality levels for consumers according to Nuernberg et al. [18].

The fatty acid composition observed for the most abundant FAs were: C14:0 at 3.54%, C16:0 at 26.69%, C18:0 at 14.98%, C16:1 cis-9 at 3.31%, C18:0 at 14.98%, C18:1 cis-9 at 37.46%, C18:2 cis-9 cis 12 at 1.60%, SFA at 47.23%, MUFA at 48.34%, and PUFA at 2.87% (Table 1). The proportion of MUFA was higher than SFA, and oleic acid (C18:1 cis-9) was the most abundant single fatty acid (37.46%).The FA composition presented in this work is similar to those reported in the literature for Nellore or other *Bos indicus* breeds [19-21]. This population also presented, in relation to *Bos taurus* breeds, average composition of fatty acids which is in agreement with reports from the USDA [22]. However, in this study a lower quantity of PUFA was observed in general, but not for C18:2 cis-9 trans-11, consequently a lower ratio of PUFA/SFA (6.08%). Similar MUFA and PUFA results have been reported in previous studies that utilized *Bos indicus* steers [20,23].

**Table 1:** Descriptive statistics, variance components and heritability by GBLUP for IMF deposition and composition in Nellore

**Trait**	**Terminology**^**1**^	**N**	**Mean ± SE**^**2**^	**Genetic variance**	**Residual variance**	**Total variance**	**h**^**2**^ **± SE**
IMF (%)	Intramuscular fat	382	2.77 ± 0.05	0.196	0.490	0.686	0.29 ± 0.16
C12:0 (mg/mg)	Lauric acid	374	0.07 ± 0.001	0.000039	0.00072	0.000761	0.05 ± 0.09
C14:0	Myristic acid	378	3.54 ± 0.03	0.0530	0.250	0.303	0.17 ± 0.11
C14:1 cis-9	Myristoleic acid	378	0.96 ± 0.01	0.0076	0.041	0.0486	0.16 ± 0.11
C15:0	Pentadecylic acid	378	0.80 ± 0.02	0.0	0.052	0.052	0 ± 0.06
C16:0	Palmitic acid	378	26.69 ± 0.15	0.6070	7.068	7.675	0.08 ± 0.10
C16:1 cis-9	Palmitoleic acid	378	3.31 ± 0.04	0.0640	0.354	0.418	0.15 ± 0.10
C17:0	Margaric acid	378	1.07 ± 0.009	0.0061	0.019	0.0251	0.24 ± 0.15
C17:1	Heptadecenoic acid	378	0.58 ± 0.007	0.0024	0.009	0.0114	0.20 ± 0.12
C18:0	Stearic acid	378	14.98 ± 0.14	1.3380	5.348	6.686	0.20 ± 0.12
C18:1 cis-9	Oleic acid	378	37.46 ± 0.22	2.0720	10.826	12.898	0.16 ± 0.11
C18:1 cis-11	Cis-Vaccenic acid	378	2.98 ± 0.05	0.0850	0.357	0.442	0.02 ± 0.09
C18:1 cis-12	Cis-12 Octadecenoic	377	0.91 ± 0.02	0.0030	0.034	0.037	0.09 ± 0.10
C18:1, cis-13	Cis-13 Octadecenoic	377	0.58 ± 0.008	0.0	0.021	0.021	0 ± 0.06
C18:1 cis-15	Cis-15 Octadecenoic	377	0.06 ± 0.002	0.0	0.0005	0.0005	0 ± 0.06
C18:1 trans-6, 7, 8	Trans-6,7,8 Octadecenoic	378	0.18 ± 0.004	0.0007	0.0055	0.0062	0.11 ± 0.09
C18:1 trans-10, 11, 12	Trans-10,11,12 Octadecenoic	378	1.07 ± 0.02	0.0236	0.0926	0.1162	0.20 ± 0.12
C18:1 trans-16	Trans-16 Octadecenoic	377	0.14 ± 0.003	0.0	0.0023	0.0023	0 ± 0.09
C18:2 cis-9 cis-12 n-6	Linoleic acid	377	1.60 ± 0.03	0.0340	0.239	0.273	0.12 ± 0.10
C18:2 cis-9 trans-11	Vaccenic acid	377	0.21 ± 0.003	0.0001	0.0026	0.0027	0.04 ± 0.09
C18:2 trans-11 cis-15	Octadecenoic acid	374	0.07 ± 0.001	0.00007	0.0004	0.00047	0.13 ± 0.11
C18:3 n-6	α-Linolenic acid	377	0.06 ± 0.001	0.00008	0.0002	0.00028	0.24 ± 0.13
C18:3 n-3	γ-Linolenic acid	376	0.16 ± 0.005	0.00026	0.0017	0.00196	0.13 ± 0.11
C20:1	Eicosanoic acid	374	0.11 ± 0.002	0.00013	0.0014	0.00153	0.09 ± 0.10
C20:2	Eicosadienoic acid	306	0.01 ± 0.0003	0.0	0.00004	0.00004	0 ± 0.08
C20:3 n-6	Eicosatrienoic acid	373	0.11 ± 0.003	0.00018	0.00235	0.00253	0.07 ± 0.09
C20:4 n-6	Arachidonic acid	377	0.36 ± 0.008	0.00221	0.0235	0.0251	0.09 ± 0.10
C20:5 n-3 (EPA)	Eicosapentaenoic acid	376	0.08 ± 0.002	0.00005	0.00135	0.00139	0.04 ± 0.09
C22:5 n-3 (DPA)	Docosapentaenoic acid	377	0.17 ± 0.003	0.00047	0.00387	0.00434	0.11 ± 0.10
C22:6 n-3 (DHA)	Docosahexaenoic acid	364	0.03 ± 0.001	0.00003	0.00018	0.00021	0.13 ± 0.10
SFA^3^	Sum of saturated FA	377	47.23 ± 0.23	1.960	15.00	16.960	0.11 ± 0.09
MUFA^3^	Sum of monounsaturated FA	377	48.34 ± 0.23	2.367	14.963	17.33	0.14 ± 0.10
PUFA^3^	Sum of polyunsaturated FA	377	2.87 ± 0.04	0.097	0.547	0.644	0.15 ± 0.10
n-3^4^	Sum of omega-3	377	0.44 ± 0.09	0.00034	0.0016	0.00194	0.17 ± 0.11
n-6^4^	Sum of omega-6	377	2.13 ± 0.04	0.00055	0.0031	0.00365	0.15 ± 0.11
PUFA:SFA	Ratio of PUFA to SFA	377	6.08 ± 0.003	0.0623	0.41	0.4723	0.13 ± 0.10
n-6:n-3	Ratio of n-6 to n-3	377	4.84 ± 0.11	0.00395	0.0231	0.02705	0.14 ± 0.10
AI^5^	Atherogenic index	377	0.82 ± 0.05	0.03943	1.0784	1.11783	0.03 ± 0.09

^1^IUPAC Compendium of Chemical Terminology.

^2^SE Standard error.

^3^MUFA, PUFA and SFA were the total of all monounsaturated, polyunsaturated and saturated fatty acids, respectively.

^4^n-3 and n-6 were total of omega 3 and 6 fatty acids.

^5^Atherogenic index = [12:0 + 4(14:0) + 16:0]/(SFA + PUFA).

The omega 6: omega 3 (n-6:n-3) ratio was high (4.84), which has a beneficial effect on patients with chronic diseases. A ratio of 4:1 has been associated with a 70% decrease in total mortality in the secondary prevention of cardiovascular disease [24]. Omega 6 and 3 fatty acids are essential cell membrane and tissue constituents that are important for many biological functions. These fatty acids are also essential for synthesis of prostaglandins, thromboxane, leukotriene, hydroxyl fatty acids, and lipoxins that are involved with inflammatory response. Humans and other mammals can convert n-6 to n-3 using desaturation enzymes but this conversion is slow and there is competition between n-6 and n-3 fatty acids for the desaturation enzymes [25].

### Heritability

Descriptive statistics and heritabilities estimated using a genomic relationship **G** matrix are in Table 1. Heritabilities estimated in this study varied from low (<0.10 for C12:0, C16:0, C18:1 cis-11, C18:1 cis-12, C18:2 cis-9 trans-11, C20:1, C20:3 n-6, C20:4 n-6, C20:5 n-3 and AI, respectively) to moderate (up to 0.29 for IMF, C14:0, C14:1 cis-9, C16:1 cis-9, C17:0, C17:1, C18:0, C18:1 cis-9, C18:1 trans-6, 7, 8, C18:1 trans-10, 11, 12, C18:2 cis 9 cis-12 n-6, C18:2 trans-11 cis-15, C18:3 n-6, C18:3 n-3, C22:5 n-3, C22:6 n-3, SFA, MUFA, PUFA, Sn-3, Sn-6 and n-6:n-3). For C15:0, C18:1 cis-13, C18:1 cis-15, C18:1 trans-16, C20:2 the heritability estimates were zero. In Angus [26] and Japanese Black cattle [27] estimates of heritability for IMF fat deposition and composition traits were higher than in this study. The lower values of heritability reported for this population could be explained by the reduced sample size [28] or lower amount of genetic variation in the population [29].

The estimates of genomic heritability from Bayes B approach (Table 2) were also low to moderate (from 0.09 to 0.46) and generally similar to values obtained in ASReml using a genomic relationship matrix.

**Table 2:** Posterior means of variance components for IMF deposition and composition in Nellore by Bayes B

**Trait**	**Genetic variance**	**Residual variance**	**Total variance**	**Genomic heritability**
IMF (%)	0.16	0.48	0.64	0.25
C12:0 (mg/mg)	0.0001	0.0006	0.0007	0.18
C14:0	0.06	0.23	0.29	0.20
C14:1 cis-9	0.03	0.02	0.05	0.25
C15:0	0.006	0.04	0.046	0.12
C16:0	2.42	5.47	7.89	0.31
C16:1 cis-9	0.10	0.30	0.40	0.24
C17:0	0.004	0.02	0.024	0.17
C17:1	0.002	0.01	0.012	0.17
C18:0	1.23	5.28	6.51	0.19
C18:1 cis-9	6.08	7.11	13.19	0.46
C18:1 cis-11	0.04	0.33	0.37	0.11
C18:1 cis-12	0.005	0.03	0.035	0.13
C18:1, cis-13	0.02	0.002	0.022	0.09
C18:1 cis-15	0.0009	0.0004	0.00049	0.17
C18:1 trans-6, 7, 8	0.0008	0.005	0.0058	0.13
C18:1 trans-10, 11, 12	0.02	0.09	0.11	0.16
C18:1 trans-6	0.0003	0.002	0.0023	0.16
C18:2 cis 9 cis-12 n-6	0.03	0.23	0.26	0.13
C18:2 cis9, trans-11	0.0003	0.002	0.0023	0.12
C18:2 trans-11 cis-15	0.0001	0.0004	0.0005	0.22
C18:3 n-6	0.00007	0.0003	0.00037	0.21
C18:3 n-3	0.0003	0.002	0.0023	0.14
C20:1	0.0002	0.001	0.0012	0.16
C20:2	0.00005	0.00002	0.00007	0.22
C20:3 n-6	0.0003	0.002	0.0023	0.14
C20:4 n-6	0.002	0.02	0.022	0.08
C20:5 n-3	0.0002	0.001	0.0012	0.17
C22:5 n-3	0.0007	0.003	0.0037	0.16
C22:6 n-3	0.00006	0.0002	0.00026	0.24
SFA^1^	1.36	15.54	16.90	0.08
MUFA^1^	1.67	15.18	16.85	0.10
PUFA^1^	0.09	0.54	0.63	0.14
n-3^2^	0.004	0.013	0.017	0.25
n-6^2^	0.0005	0.003	0.0035	0.15
PUFA:SFA	0.003	0.02	0.023	0.11
n-6:n-3	0.66	1.23	1.89	0.34
AI^3^	0.007	0.03	0.037	0.16

^1^MUFA, PUFA and SFA were the total of all monounsaturated, polyunsaturated and saturated fatty acids, respectively.

^2^n-3 and n-6 were total of omega 3 and 6 fatty acids.

^3^Atherogenic index = [12:0 + 4(14:0) + 16:0]/(SFA + PUFA).

### Genome wide association studies and genomic regions identified

GWAS and genomic regions identified were reported for those traits that showed genomic heritability ≥ 0.10: IMF, 33 different FA and ratios, and one FA index. The 1-Mb SNP window regions that explained more than 1% of the genetic variance were used to search for putative candidate genes (PCG) and are presented on Table 3. Therefore, 35 traits were used for GWAS and these represented 23 different 1-Mb genomic regions (Table 3). These regions were distributed over 12 different chromosomes: 2, 3, 6, 7, 8, 9, 10, 11, 12, 17, 26 and 27 and the corresponding PCG in these regions are reported in Table 3. Intramuscular fat was one of the traits that presented moderate heritability (0.25), however no region that explained more than 1% of the genetic variance for this trait was identified.

**Table 3:** QTL regions associated with fatty acid composition in Nellore steers by Bayes B

**Group**	**Traits**	**QTL window (first and last SNP)**	**Number of SNP /window**^**1**^	**%Variance explained SNP window**^**1**^	**Chr**	**Map position (UMD 3.1 bovine assembly)**	**PCG**^**2**^
**Saturated fatty acid**	C14:0	rs43328164 - rs134696015	295	1.06	3	6009105 - 6999643	*-*^**3**^
	C18:0	rs133274959 - rs136576856	213	3.46	3	25008982 - 25990012	*HMGCS2, PHGDH, HSD3B1, HAO2*
	C16:0	rs134160160 - rs109942510	285	1.38	3	26002777 - 26998679	*WARS2*
	C14:0	rs41627556 - rs110731616	332	1.43	9	36013595 - 36997263	*GNG11, RGS5*
	C18:0	rs136405986 - rs137105475	114	1.08	10	74001100 - 74957582	*DHRS7*
	C18:0	rs132804279 - rs109645596	102	2.08	11	105009792 - 105985714	*NUP214*
	C16:0	rs109773631 - rs135618512	277	1.53	12	13000697 - 13997298	-
	C12:0	rs42924061 - rs134942057	214	3.48	12	60000226 - 60994461	*SLITRK6*
**Monounsat. fatty acid**	C14:1 cis-9	rs137683417 - rs29003360	232	1.86	2	26002605 - 26999718	*GAD1, Sp5*
	C18:1 cis-9	rs134160160 - rs109942510	285	1.40	3	26002777 - 26998679	*WARS2*
	C16:1 cis-9	rs133274959-rs136576856	213	1.42	3	25008982 - 25990012	*HMGCS2, PHGDH, HSD3B1, HAO2*
	C18:1 cis-9	rs133274959-rs136576856	213	2.57	3	25008982 - 25990012	*HMGCS2, PHGDH, HSD3B1, HAO2*
	C18:1 t-16	rs133728493 - rs109630757	264	2.56	6	50008629 - 50996673	-
	C16:1cis-9	rs135892505 - rs109693564	184	1.04	7	80006438 - 80997126	*-*
	C16:1cis-9	rs136405986 - rs137105475	114	1.18	10	74001100 - 74957582	*DHRS7*
	C14:1cis-9	rs110517663 - rs137137362	127	1.43	11	27000446 - 27993515	*ABCG5*
	C18:1 cis-9	rs109773631 - rs135618512	277	1.91	12	13000697 - 13997298	*-*
	C14:1 cis-9	rs137754875 - rs135670282	203	1.55	12	66002783 - 66997356	*GPC6*
	C18:1 t-16	rs109872854 - rs134277482	250	2.17	17	41001273 - 41991683	*RAPGEF2, RPS27*
**Polyunsat. fatty acid**	C22:5 n-3	rs132839318 - rs134264692	306	3.35	3	27001604 - 27997941	*SPAG7, WDR3*
	C18:2 t-11c-15	rs42404785 - rs133183089	144	1.69	3	72000121 - 72997801	*AQP7, LOXL2*
	C18:3 n-6	rs109612389 - rs133661384	205	1.34	7	34004443 - 34998434	-
	C18:2 t-11c-15	rs135377389 - rs133297940	267	3.49	8	68013821 - 68993778	-
	C22:5 n-3	rs110411459 - rs137802105	202	4.74	10	29000222 - 29962997	*-*
	C20:5 n-3	rs137823965 - rs42507195	199	2.19	10	49005824 - 49980174	*RORA*
	C18:3 n-3	rs42582725 - rs133775322	132	1.33	17	24002089 - 24987264	-
	C18:2 t-11c-15	rs137602675 - rs133056879	187	1.01	26	20001875 - 20999270	-
	C20:3 n-6	rs134302284 - rs136338106	144	1.77	27	26000833 - 26975692	-
**Total of MUFA**	MUFA	rs133274959 - rs136576856	213	3.24	3	25008982 - 25990012	*HMGCS2, PHGDH, HSD3B1, HAO2*
		rs134160160 - rs109942510	285	1.13	3	26002777 - 26998679	*WARS2*
**Total of n-3**	n-3	rs110411459 - rs137802105	202	2.59	10	29000222 - 29962997	-
		rs137823965 - rs42507195	199	2.25	10	49005824 - 49980174	-
		rs1322839318 - rs134264692	306	1.37	3	27001604 - 27997941	*SPAG7, WDR3*
**Total of n-6**	n-6	rs134302284 - rs136338106	144	2.47	27	26000833 - 26975692	*-*

^1^1 Mb window.

^2^Positional candidate genes.

^3^No putative candidate gene associated with the trait.

### Saturated fatty acids

Eight genomic regions (1 Mb windows) explained more than 1% of genotypic variation for C12:0, C14:0, C16:0, and C18:0 (Table 3). These regions overlap with QTL previously reported for marbling score [28], backfat thickness [29], carcass weight and body weight in Angus cattle [30]. Additional file 1 shows in more detail the Manhattan plot of the proportion of genetic variance explained by window across the 29 autosomes for the important saturated fatty acids for beef palatability: C12:0 (lauric acid), palmitic acid (C16:0), stearic acid (C18:0).

BTA12 at 60 Mb QTL region was associated with C12:0 fatty acid and harbors the PCG *SLIT* and *NTRK*-like family, member 6 (*SLITRK6*). This gene is related to the lipopolysaccharide receptor complex (GO:0016021). Lipopolysaccharide (endotoxin) is an important component of gram-negative cell walls, which can start the inflammatory process by binding to the *SLITRK6* complex. This complex is located at the surface of innate immune cell, which presents an important role in obesity associated with insulin resistance [31].

BTA3 at 6 Mb and BTA9 at 36 Mb were associated with C14:0 fatty acid. In the first region, no PCG was identified. In the second region, the following PCGs were identified: guanine nucleotide binding protein (G protein), gamma 11 (*GNG11*) and regulator of G-protein signaling 5 (*RGS5*). These genes are associated with G proteins that have been implicated in the regulation of body weight and metabolic function, hyperinsulinemia, impaired glucose tolerance and resistance to insulin in mice [32].

BTA3 at 26 Mb and BTA12 at 13 Mb were associated with C16:0 fatty acid. In the first QTL region the tryptophanyl tRNA synthetase 2, mitochondrial (*WARS2*) gene was identified. In BTA12 at 13 Mb no PCG was identified. *WARS2* gene encodes an essential enzyme that catalyzes aminoacylation of tRNA with tryptophan. The *WARS2* protein contains a signal peptide for mitochondrial import, OMIM: 604733 [33], and is involved with regulation of fat distribution in human visceral and subcutaneous fat [34].

BTA3 at 25 Mb, BTA10 at 74 Mb and BTA11 at 105 Mb QTL regions were associated with C18:0 fatty acid. On BTA3 at 25 Mb four PCG were identified: 3-hydroxy-3-methylglutaryl-Coenzyme A synthase 2, mitochondrial (*HMGCS2*), phosphoglycerate dehydrogenase (*PHGDH*), hydroxy-delta-5-steroid dehydrogenase, 3 beta- and steroid delta-isomerase 1 (*HSD3B1*) and hydroxyacid oxidase 2, long chain (*HAO2*). *HMGCS2* was associated with fatty acid oxidation and ketogenesis in HepG2 cells [35] and is directly regulated by *PPAR-α* gene that is an important key regulator of β-oxidation. On the other hand the *PPAR-γ* expression induces lipogenesis by PXR activation in mice liver [36]. *PHGDH* is related to the regulation of gene expression according to gene ontology (GO) terms (GO:0010468), *HSD3B1* gene is associated with steroid biosynthesis (GO:0006694) and metabolic process (GO: 0008202) and *HAO2* is associated with fatty acid oxidation (GO:0019395).

On BTA10 at 74 Mb QTL region, dehydrogenases/reductases (*SDRs*) (*DHRS7*) was identified. *DHRS7* catalyzes the oxidation/reduction of a wide range of substrates, including retinoid and steroids [37] and has high expression level in adipocyte and skeletal muscle [38]. In addition, this gene is responsible for the final step in cholesterol production, the conversion of 7-dehydrocholesterol to cholesterol [39].

On BTA11 at 105 Mb region, *NUP214* was identified. This gene is involved with intermembrane transport. The nucleoporins are characterized by phenylalanine-glycine rich (*FG*) repeat sequences and the *FG* domains have an unfolded structure and are responsible for interaction with importin-cargo complexes that move through the pore. One of these importin-cargo complexes is sterol regulatory element binding proteins-sterol-sensing accessory factor (*SREBP-SCAP*). This factor enters the nucleus, then binds to sterol regulatory elements (*SRE*) in the promoter regions of genes, whose products mediate the synthesis of cholesterol and fatty acids [40].

### Monounsaturated fatty acids

**Figure 1:**
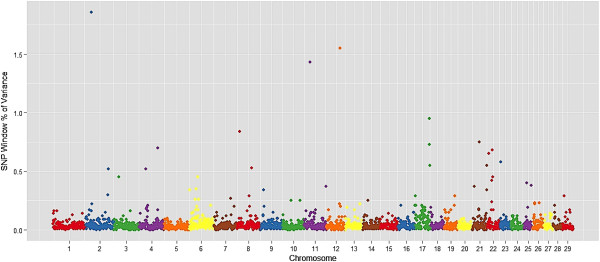
**Manhattan plot of the genome-wide association study for C14:1 cis 9 (myristoleic acid) in Nellore.** The X-axis represents the chromosomes, and the Y-axis shows the proportion of genetic variance explained by SNP window from Bayes B analysis.

**Figure 2:**
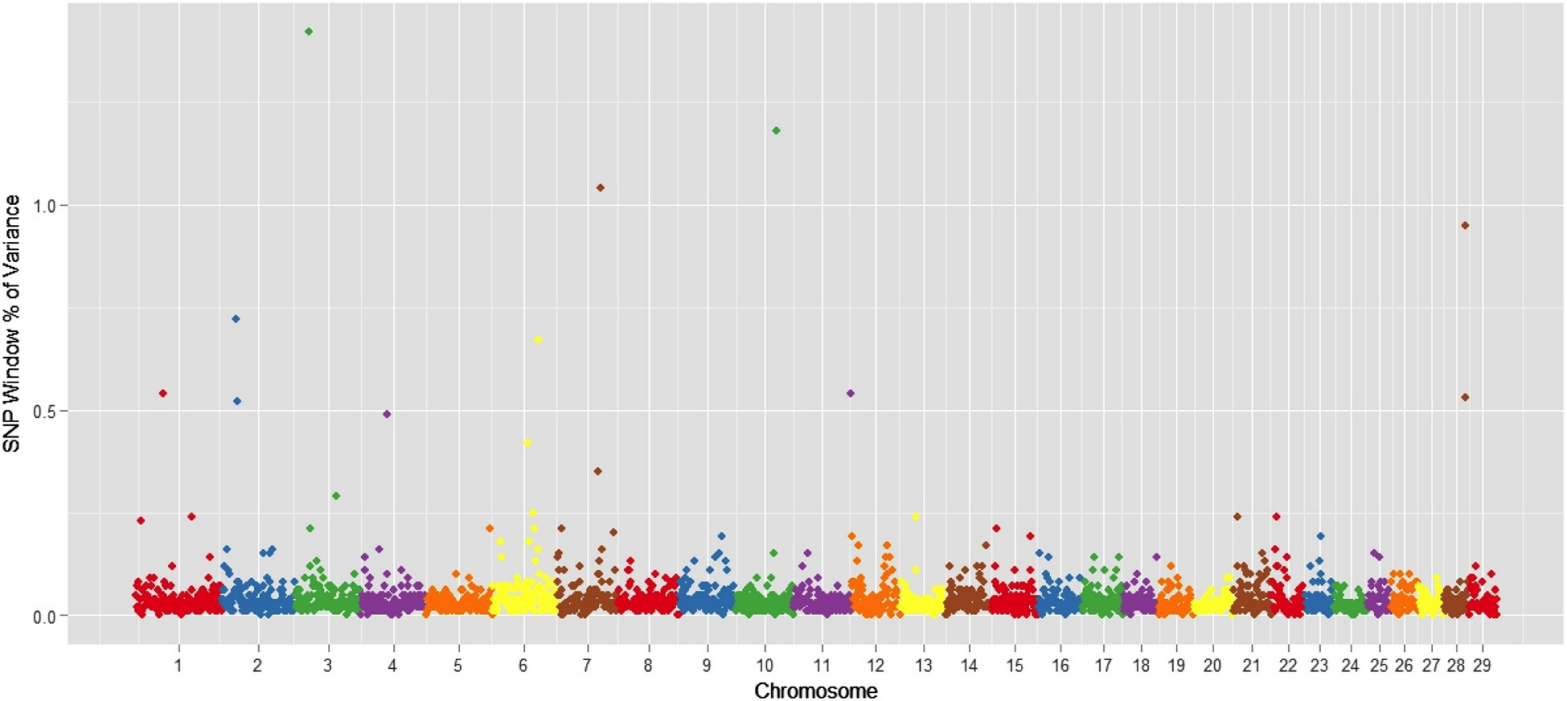
**Manhattan plot of the genome-wide association study result for C16:1 cis9 (palmitoleic acid) in Nellore.** The X-axis represents the chromosomes, and the Y-axis shows the proportion of genetic variance explained by SNP window from Bayes B analysis.

**Figure 3:**
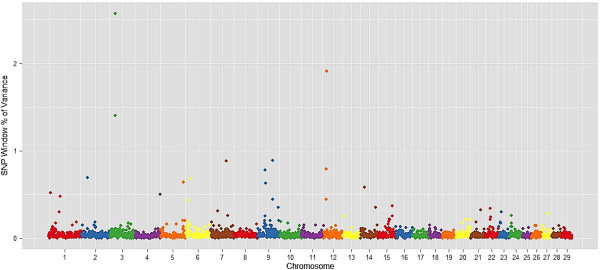
**Manhattan plot of the genome-wide association study result for C18:1 cis9 (oleic acid) in Nellore.** The X-axis represents the chromosomes, and the Y-axis shows the proportion of genetic variance explained by SNP window from Bayes B analysis.

Ten genomic regions (1 Mb region) explained more than 1% of genotypic variation for monounsaturated fatty acids, which relates C14:1 cis-9, C16:1 cis-9, C18:1 cis-9, and C18:1 trans-16 (Table 3). These regions overlap with QTL reported for carcass weight, marbling score in Angus [30], docosahexaenoic acid content in Charolais x Holstein crossbred cattle [41] and palmitoleic acid in dairy cattle [42]. Manhattan plots of the proportion of genetic variance explained by each 1-Mb window (2,527 windows) across the 29 autosomes for the most important fatty acid for beef quality and human health: myristoleic acid (C14:1 cis-9), palmitoleic acid (C16:1 cis-9), and oleic acid (C18:1 cis-9) and are in Figures 1, 2 and 3, respectively.

BTA2 at 26 Mb, BTA11 at 27 Mb , and BTA12 at 66 Mb were associated with C14:1 cis-9 fatty acid. On BTA2 at 26 Mb region, glutamate decarboxylase 1 (*GAD1*) and specificity protein 5-transcription factor (*Sp5*) were identified. The *GAD1* gene is involved with food behavior and insulin secretion. It was shown to be associated with morbid obesity [43] in humans, and body weight and daily gain in cattle [44]. *Sp5* transcription factors are involved in the regulation of pyruvate kinase, lactate dehydrogenase and fatty acid synthase in cancer cells [45,46]. On BTA11 at 27 Mb region, ATP-binding cassete, sub-family G (WHITE), member 5 (*ABCG5*) was identified. The *ABCG* family members were associated with cellular lipid-trafficking in macrophages and hepatocytes. This gene also presents an important role in the *PPARγ* and LXR pathways [47]. On BTA12 at 66 Mb region, glypican 6 (*GPC6*) was identified. Glypicans family are involved with the control of cell growth and cell division. Other glypican gene, *GPC4* was associated with body fat distribution and obesity in humans [48]. A previous GWAS using chicken population reported an association between *GPC6* and body weigh, which has a stronger correlation with body fatness and obesity [49].

BTA3 at 25 Mb, BTA7 at 80 Mb and BTA10 at 74 Mb were associated with C16:1 cis-9 fatty acid. Both BTA3 at 25 Mb and BTA10 at 74 Mb regions were also associated with saturated fatty acids, where *HMGCS2, PHGDH, HSD3B1, HAO2,* and *DHRS7* were described above. On BTA7 at 80 Mb region, no PCG associated with this trait was identified.

BTA3 at 25 Mb, BTA3 at 26 Mb, and BTA12 at 13 Mb were associated with C18:1 cis-9. The first two regions were also associated with saturated fatty acids (C18:0 and C16:0, respectively) and C16:1 cis-9. On BTA12 at 13 Mb region, there were no annotated genes [50].

BTA6 at 50 Mb and BTA17 at 41 Mb were associated with C18:1 trans-16. On BTA 6 at 50 Mb region, no PCG associated with this trait was identified, and on BTA17 at 41 Mb region one PCG was found: Rap guanine nucleotide exchange factor (*RAPGEF2*), that selectively and non-covalently interacts with diacylglycerol, a diester of glycerol and two fatty acids (GO:0019992).

Four genomic regions were associated with saturated and monounsaturated fatty acids. BTA3 at 26 Mb and BTA12 at 13 Mb were associated with C16:0 and C18:1 cis-9. BTA3 at 25 Mb was associated with C18:0 and C18:1 cis-9. BTA3 at 25 and BTA10 at 74 Mb were associated with C18:0 and C16:1 cis-9. We also observed a negative phenotypic correlation between C16:0 and C18:1 (r = -0.65) and between C18:0 and C18:1(r = -0.72). Palmitic acid (C16:0) carbon chain skeleton is a predominant source for fatty acid elongation and desaturation, which might generate palmitoleic (C16:1 cis-9), stearic (C18:0), and oleic (C18:1 cis-9) fatty acids [51]. Examination of the 30 markers with the larger effect in regions of BTA3 at 26 Mb and BTA12 at 13 Mb (Table 4) and BTA3 at 25 Mb (Additional file 2) revealed that alleles positively associated with saturated fatty acid were negatively associated with unsaturated fatty acids. This indicates that QTL in these regions are directly or indirectly involved with elongation and/or desaturation.

**Table 4:** Top 30 markers effect in BTA3 at 26 Mb and BTA12 at 13 Mb QTL associated with C16:0 and C18:1 cis-9 in Nellore steers

**BTA3 at 26 Mb**				**BTA12 at 13 Mb**			
**SNP name**	**Marker effect C16:0**	**Marker effect 18:1 cis-9**	**Position (Chr_Bp)**	**SNP name**	**Marker effect C16:0**	**Marker effect 18:1 cis-9**	**Position (Chr_bp)**
rs136571109	1.49E-02	-8.42E-03	3_26426591	rs109310464	1.53E-02	-3.10E-02	12_13152910
rs110996601	1.45E-02	-6.58E-03	3_26425542	rs137546638	1.97E-03	-6.73E-03	12_13172924
rs134173038	1.40E-02	-6.05E-03	3_26427414	rs110182797	1.01E-03	-7.53E-04	12_13254936
rs137302352	4.10E-03	-2.29E-03	3_26400703	rs110951319	6.73E-04	-4.73E-04	12_13257070
rs135909241	9.74E-04	-2.55E-04	3_26446293	rs109014250	5.98E-04	-6.22E-04	12_13687406
rs109610305	7.91E-04	-1.72E-05	3_26497009	rs137052772	5.83E-04	-3.95E-04	12_13343491
rs137775131	5.12E-04	-7.50E-05	3_26501617	rs42625291	5.42E-04	-1.25E-03	12_13689345
rs109564367	5.01E-04	-8.52E-03	3_26073307	rs132782691	5.23E-04	-1.87E-04	12_13334837
rs110447932	4.30E-04	-8.35E-03	3_26072388	rs110645603	2.15E-04	-6.03E-05	12_13226418
rs109719131	3.84E-04	-9.90E-05	3_26465447	rs135746294	1.74E-04	-2.71E-05	12_13037523
rs110569793	3.62E-04	-9.64E-05	3_26476929	rs109497621	1.16E-04	-5.02E-05	12_13679288
rs133474163	3.39E-04	-1.56E-02	3_26143362	rs42625298	1.13E-04	-1.40E-04	12_13703328
rs110930607	3.22E-04	-1.90E-04	3_26332392	rs133583231	1.09E-04	-5.69E-05	12_13557660
rs134881809	3.10E-04	-2.36E-04	3_26505695	rs110721694	1.01E-04	-2.78E-03	12_13696926
rs111020805	2.70E-04	-1.92E-03	3_26108986	rs135841134	9.62E-05	-4.91E-05	12_13897680
rs134887465	2.68E-04	-2.04E-04	3_26385596	rs41667835	9.59E-05	-1.41E-04	12_13710147
rs110953593	2.61E-04	-8.47E-03	3_26075185	rs136432856	9.51E-05	3.08E-05	12_13493245
rs109658959	2.57E-04	-7.53E-03	3_26596824	rs133513235	9.45E-05	-4.21E-04	12_13251749
rs136570164	2.56E-04	-7.23E-05	3_26961906	rs137549822	8.60E-05	-6.65E-04	12_13081160
rs132696738	2.51E-04	-2.80E-04	3_26338744	rs110015470	8.45E-05	-3.18E-04	12_13682141
rs134559574	2.46E-04	-3.01E-05	3_26774155	rs133443666	8.30E-05	-1.46E-04	12_13520963
rs133573311	2.20E-04	-1.54E-04	3_26330114	rs208626835	8.10E-05	5.29E-06	12_13515819
rs135402139	1.94E-04	-1.26E-04	3_26954008	rs134142865	7.61E-05	-2.40E-04	12_13264826
rs135422840	1.71E-04	-3.00E-03	3_26020004	rs134240141	7.39E-05	-7.90E-05	12_13571494
rs136944072	1.66E-04	-2.63E-03	3_26149734	rs110659649	6.83E-05	-3.57E-05	12_13092209
rs110497471	1.56E-04	-2.50E-04	3_26576223	rs135136417	6.58E-05	-3.14E-05	12_13266029
rs137361087	1.45E-04	-3.15E-03	3_26144230	rs109064784	6.32E-05	-8.96E-05	12_13785143
rs110049045	1.37E-04	-7.04E-05	3_26975095	rs135831828	6.31E-05	-3.94E-05	12_13769919
rs133610187	1.36E-04	-2.31E-04	3_26290060	rs135423477	6.25E-05	-1.43E-04	12_13642422
rs110819650	1.35E-04	-4.69E-05	3_26979811	rs109147565	6.16E-05	-1.53E-03	12_13097096

### Polyunsaturated fatty acids

Nine genomic regions (1 Mb window) explained > 1% of genotypic variation for group of polyunsaturated fatty acids, which relates C18:2 cis-9 cis12 n-6, C18:2 trans-11 cis-15, C18:3 n-3, C18:3 n-6, C20:3 n-6, C20:5 n-3, and C22:5 n-3 (Table 3). These overlapped with QTL reported for marbling score, body weight in Angus cattle [30], intramuscular fat, saturated fatty acid content, stearic acid in Fleckvieh bulls [52] and RFI in half-sib families from Angus and Charolais [53]. Additional file 3 shows in more detail the Manhattan plot of the proportion of genetic variance explained by window across the 29 autosomes for the important fatty acid to human health: γ-linolenic acid (C18:3 n-3), α-linolenic acid (C18:3 n-6) and total of n-3 (n-3).

BTA3 at 72 Mb, BTA 8 at 68 Mb, and BTA26 at 20 Mb were associated with C18:2 trans-11 cis-15 fatty acid. On BTA3 at 72 Mb QTL region, two PCG related to lipid metabolism were observed: aquaporin 7 (*AQP7*) and lysil oxidase-like2 (*LOXL2*). *AQP7* is involved with the *PPAR* signaling pathway [54], while *LOXL2* is associated with lean body mass in mouse [55]. On BTA 8 at 68 Mb and BTA26 at 20 Mb no PCG was associated with this trait.

BTA10 at 49 Mb region was associated with C20:5 n-3 fatty acid, which harbors the *RAR*-related orphan receptor A (*RORA*) gene that is related to steroid hormone receptor activity. When combined with a steroid hormone, it produces the signal within the cell to initiate a change in cell activity or function (GO:0003707).

BTA3 at 27 Mb and BTA10 at 29 Mb QTL regions explained more than 1% of genetic variance for C22:3 n-3. The first region (BTA3 at 27 Mb) harbors two PCG: *SPAG17* and *WDR3*. These genes are involved with nucleus membrane (cellular components), the lipid bilayers that surround the nucleus and that form the nuclear envelope excluding the intermembrane space (GO:0031965). In the second region no PCG involved with this trait was identified. In other regions associated with C18:3 n-3 (BTA7 at 34 Mb), C18:3 n-6 (BTA17 at 24 Mb), and C20:3 n-6 (BTA27 at 26 Mb) no annotated genes were found [56].

The total of omega-3 (n-3) and omega-6 (n-6) GWAS result is consistent with individual polyunsaturated n-3 and n-6 fatty acids, where the same QTL regions were associated with these traits (BTA3 at 27 Mb and BTA 27 at 26 Mb).

GWAS have been used to investigate complex traits in many species including livestock [6]. Deposition and composition of fat in mammalians are complex traits and are influenced by many loci throughout the genome [57]. GWAS provides one approach to understand the genetic variation in complex traits, by identifying regions that can be fine-mapped to identify individual loci responsible for variation [58]. In the present GWAS two interesting QTL regions BTA3 at 25 Mb and BTA3 at 26 Mb were associated with saturated, monounsaturated, and polyunsaturated fatty acids in *Bos indicus* cattle. These regions harbor interesting PCG, which are involved with lipid metabolism in different species.

Previous studies reported CCAAT/enhancer binding proteins (*C/EBPβ*), peroxisome proliferator-activated receptor gamma (*PPARγ* or *PPARG*), carnitine palmitoyltransferase–1 beta (*CPT–1β*), stearoylcoenzyme A desaturase (*SCD*), AMP-activated protein kinase alpha (*AMPKα*), and G-coupled protein receptor 43 (*GPR43*) genes to be related to fat deposition and fatty acid composition in Angus [59]. In this GWAS and in a recent GWAS publication using Angus cattle, the regions that harbor these genes were not associated with fat deposition and fatty acid composition [60]. However, the positional candidate genes *HMGCS2, PHGDH, HSD3B1 and HAO2* identified in the present study are involved in the *PPAR* signaling pathway in human (PATH: hsa03320), carbon metabolism (PATH:bta01200), lipid metabolism and steroid hormone biosynthesis (PATH: bta00140), carbohydrate metabolism and glycosylate and decarboxylase metabolism (PATH: bta00630) according to Kegg: Kyoto Encyclopedia of Genes and Genomes [61], respectively.

This is the first GWAS for intramuscular fat deposition and composition in Nellore. GWAS results in *Bos taurus* (Angus and Japanese Black cattle) reported different genomic regions associated with fat deposition and composition than those reported herein [62,63]. Previously, it has been reported that a QTL on BTA19 was associated with fatty acid composition in *Bos taurus* breeds. This region on BTA19 harbors fatty acid synthase gene (*FASN*), which is an enzyme involved with *de novo* synthesis of long-chain fatty acid in mammalian, lipogenesis. *FASN* has been suggested as a candidate gene for fat traits in beef cattle [60,62,63].

Differences in SNP allele frequencies and linkage disequilibrium profile (LD between SNPs and causal variants) may explain the different marker effects between *Bos indicus* and *Bos taurus* cattle [64,65]. This explanation has been confirmed by Bolormaa and collaborators [66], who compared marker effects using GWAS for *Bos taurus* and *Bos indicus* animals, which demonstrated that a SNP effect depends on the origin of alleles and the QTL segregation. The QTL segregation could result from mutation lost or fixation of alleles in one of the breeds, and also that the mutations occurred after divergence of these breeds [67]. Furthermore, it is possible that the differences in physiological and metabolism factors could contribute to the observed differences between different breeds [12].

## Conclusion

The present study using BovineHD BeadChip (770 k) identified several 1-Mb SNP regions and genes within these regions that were associated with IMF and FA. The values of genomic heritabilities described in this study have not been reported before for Nellore cattle, and this information is important for breeding programs interesting in improving these traits. IMF deposition and composition are considered complex traits (polygenic) and are moderately heritable. In this study it is apparent that IMF composition are affected by many loci with small effects. Identification of several genomic regions and putative positional genes associated with lipid metabolism reported here should contribute to the knowledge of the genetic basis of IMF and FA deposition and composition in Nellore cattle (*Bos indicus* breed) and lead to selection for those traits to improve human nutrition and health.

## Methods

Animals were handled and managed according to Institutional Animal Care and Use Committee Guidelines from Brazilian Agricultural Research Corporation – EMBRAPA approved by the president, Dr. Rui Machado.

### Animals and phenotypes

Nellore steers (386) bred in the Brazilian Agricultural Research Corporation (EMBRAPA/Brazil) experimental breeding herd between 2009 and 2011 were available for this study. Steers were sired by 34 unrelated sires, and were selected to represent the main genealogies used in Brazil according to the National Summary of Nellore produced by the Brazilian Association of Zebu Breeders (ABCZ) and National Research Center for Beef Cattle. Animals were raised in feedlots under identical nutrition and handling conditions until slaughter at an average age of 25 months [68]. Steaks (2.54 cm thick) from the *Longissimus dorsi* muscle between the 12th and 13th ribs were collected 24 hours after slaughter.

Muscle samples (~100 g) were lyophilized and ground for IMF and FA analysis. The IMF was obtained using an Ankom XT20 extractor as described [69]. FA analysis was conducted as described by Hara and Radin [70], except the hexane to propanol ratio was increased to 3:2. Approximately 4 g of LD muscle was lyophilized, ground in liquid nitrogen, mixed with 28 mL of hexane/propanol (3:2 vol/vol) and homogenized for 1 min. Samples were vacuum filtered and 12 ml sodium sulfate (67 mg mL^− 1^) solution was added and agitated for 30 s. The supernatant was transferred to a tube with 2 g of sodium sulfate and insufflated with N_2_, after which the tube was sealed and incubated at room temperature for 30 min. Subsequently, the liquid was transferred to 10 mL test tube, insufflated with N_2_, sealed and kept at − 20°C until dry with N_2_ for methylation. The extracted lipids were hydrolyzed and methylated as described by Christie [71], except that hexane and methyl acetate were used instead of hexane:diethyl ether:formic acid (90:10:1). Around 40 mg of lipids were transferred to a tube containing 2 mL of hexane. Subsequently, 40 μL of methyl acetate were added, the sample agitated, and 40 μL of methylation solution (1.75 mL of methanol/0.4 mL of 5.4 mol/L of sodium metoxyde) were added. This mixture was agitated for 2 min and incubated for 10 min at room temperature. Then 60 μL of finishing solution (1 g of oxalic acid/30 mL of diethyl ether) were added and the mixture was agitated for 30 s, after which 200 mg of calcium chloride was added. The sample was then mixed and incubated at room temperature for 1 h. Samples were centrifuged at 3200 rpm, for 5 min at 5°C. The supernatant was collected for determination of fatty acids. Fatty acid methyl esters were quantified with a gas chromatograph (ThermoFinnigan, Termo Electron Corp., MA, USA) equipped with a flame ionization detector and a 100 m Supelco SP-2560 (Supelco Inc., PA, USA) fused silica capillary column (100 m, 0.25 mm and 0.2 μm film thickness). The column oven temperature was held at 70°C for 4 min, then increased to 170°C at a rate of 13°C min^−1^, and subsequently increased to 250°C at a rate of 35°C min^−1^, and held at 250°C for 5 min. The gas fluxes were 1.8 mL min^−1^ for carrier gas (He), 45 mL min^−1^ for make-up gas (N_2_), 40 mL min^−1^ for hydrogen, and 450 mL min^−1^ for synthetic flame gas. One μL sample was analyzed. Injector and detector temperatures were 250 and 300°C, respectively. Fatty acids were identified by comparison of retention time of methyl esters of the samples with standards of fatty acids butter reference BCR-CRM 164, Anhydrous Milk Fat-Producer (BCR Institute for Materials and Reference Measurements) and also with commercial standard for 37 fatty acids Supelco TM Component FAME Mix (cat 18919, Supelco, Bellefonte, PA). The nomenclature of fatty acids follow IUPAC Compendium [72]. Fatty acids were quantified by normalizing the area under the curve of methyl esters using Chromquest 4.1 software (Thermo Electron, Italy). Fatty acids were expressed as a weight percentage (mg/mg). These analyses were performed at the Animal Nutrition and Growth Laboratory at ESALQ, Piracicaba, São Paulo, Brazil.

### DNA extraction and genotypic data

DNA was isolated from blood as described by Tizioto et al. [68]. Genotyping was performed at the Bovine Functional Genomics Laboratory ARS/USDA and Genomics Center at ESALQ, Piracicaba, São Paulo, Brazil using BovineHD 770 k BeadChip (Infinium BeadChip, Illumina, San Diego, CA) according to manufacturer’s protocol. Genotypes were obtained in Illumina A/B allele format and used to represent a covariate value at each locus coded as 0, 1, or 2, representing the number of B alleles. Missing genotypes, represented < 0.2% of genotypes and were replaced with the average covariate value at that locus. Initial visualization and data analysis was performed by GenomeStudio Data Analysis Software [73]. The SNPs with call rate ≤ 95%, minor allele frequency (MAF) ≤ 5%, those located on sex chromosomes and those not mapped in the *Bos taurus* UMD 3.1 assembly were removed. After filtering, a total of 449,363 SNP were utilized in GWAS.

### Descriptive statistics and heritability

Descriptive statistics for IMF and FA were estimated using PROC MEANS and normality tests were performed using PROC UNIVARIATE in SAS (Ver. 9.3; SAS Inst. Cary, NC). SAS PROC MIXED was used to test independent sources for significance. Fixed effects included contemporary group classes (animals with the same origin, birth year and slaughter date) and hot carcass weight as a covariate. Animal and residuals were fitted as random effects. Restricted maximum likelihood was used to estimate animal and residual variance components, heritability and standard error (SE) using ASREML software [74]. The model used in single-trait analyses of all traits was, *y* = X*b* + Z*u* + *e*, where **y** is the vector of observations representing the trait of interest (dependent variable), **X** and **Z** are the design or incidence matrices for the vectors of fixed and random effects in **b** and **u**, respectively, and **e** was the vector of random residuals. The variance of vector **u** was **G**σ^2^_m_ for the genomic analyses where **G** is the genomic relationship matrix derived from SNP markers using allele frequencies as suggested by VanRaden [75], with σ^2^_m_ being the marker-based additive genetic variance.

### Genome wide association study

Associations between SNP and phenotypes (IMF and FA) were obtained using Bayes B, which analyzed all SNP data simultaneously and assumed a different genetic variance for each SNP locus [76,77]. The prior genetic and residual variances were estimated using Bayes C [78], with π being 0.9997. The model equation was:

$$y=\mathrm{Xb}+\sum_{j=1}^{k}a_{j}\beta_{j}\delta_{j}+e,$$

where **y** was the vector of the phenotypic values, **X** was the incidence matrix for fixed effects, **b** was the vector of fixed effects defined above, *k* was the number of SNP loci (449,363), **a**_*j*_ was the column vector representing the SNP covariate at locus *j* coded as the number of B alleles, *β*_*j*_ was the random substitution effect for locus *j*, which conditional on σ^2^_*β*_ was assumed to be normally distributed *N* (0, σ^2^_*β*_ ) when *δ*_*j*_*=* 1 but *β*_*j*_*=* 0 when *δ*_*j*_*=* 0, with *δ*_*j*_ being a random 0/1 variable indicating the absence (with probability π) or presence (with probability 1-π) of locus *j* in the model, and **e** was the vector of the random residual effects assumed normally distributed *N* (0, σ^2^_*e*_ ). The variance σ^2^_*β*_ (or σ^2^_*e*_) was a *priori* assumed to follow a scaled inverse Chi-square with *v*_*β*_*=* 4 (or *v*_*e*_*=* 10) degrees of freedom and scale parameter *S*^2^_*β*_ (or *S*^2^_*e*_). The scale parameter for markers was derived as a function of the assumed known genetic variance of the population, based on the average SNP allele frequency and number of SNP assumed to have nonzero effects based on parameter π being 0.9997. This procedure used GenSel software [8] to obtain the posterior distributions of SNP effects using Markov chain Monte Carlo (MCMC). This comprised a burn-in period of 1,000 iterations from which results were discarded, followed by 40,000 iterations from which results were accumulated to obtain the posterior mean effect of each SNP. In the Bayesian variable selection multiple-regression models with π = 0.9997 about 100-150 SNP markers were fitted simultaneously in each MCMC iteration. Inference of associations in these multiple-regression models was based on 1-Mb genomic windows rather than on single markers [8,79]. Genomic windows were constructed from the chromosome and base-pair positions denoted in the marker map file [8] based on UMD3.1 bovine assembly.

The SNP effects from every 40^th^ post burn in iteration were used to obtain samples from the posterior distribution of the proportion of variance accounted for by each window from 1,000 MCMC samples of genomic merit for each animal following Onteru et al. [79] and Peters et al. [7]. In the present study there were 2,527 1 Mb SNP windows across the 29 autosomes. The proportion of genetic variance explained by each window in any particular iteration was obtained by dividing the variance of window BV by the variance of whole genome BV in that iteration. The window BV was computed by multiplying the number of alleles that represent the SNP covariates for each consecutive SNP in a window by their sampled substitution effects in that iteration.

All traits were used for GWAS (Additional file 4) and genomic heritability estimate, but only the ones with genomic heritability ≥ 0.10 were reported. Fatty acids were indexed as groups of saturated, monounsaturated, polyunsaturated fatty acid, total of saturated fatty acid (SFA), total monounsaturated (MUFA), total of polyunsaturated (PUFA), total of omega 3 (n-3) and total of omega 6 (n-6). Genome windows with the highest posterior mean proportion of genetic variance ≥1% were considered the most important regions associated with the traits, and were declared the most promising QTL regions.

Positional candidate genes were investigated for 1 Mb windows using the Cattle Genome Browser [50] and UCSC Genome Browser [80], which allowed visualization of SNP based on *Bos taurus* genome assembly UMD 3.1. Animal QTL database (Animal QTLdb) was used to search for published QTL and trait mapping data [56]. Gene annotations were retrieved from Ensembl Genes 71 Database using Biomart software [54,81]. The functional classification of genes was done using DAVID [54] and BioGPS [38] online annotation databases. Those genes reported to be involved in fatty acid and lipid metabolism were selected as positional candidate genes.

### Availability of supporting data

The data sets supporting the results of this article are included within the article and its additional files.

## Abbreviations

IMF: Intramuscular fat; LDL: Low density lipoprotein; MUFA: Total of monounsaturated fatty acid; FA: Fatty acid; SNP: Single nucleotide polymorphism; GWAS: Genome-wide association study; QTL: Quantitative trait loci; SFA: Saturated fatty acid; PUFA: Total of polyunsaturated fatty acid; CLA: Conjugated linoleic acid; PCG: Putative candidate gene; GO: Gene ontology; MAF: Minor allele frequency; SE: Standard error; MCMC: Markov chain Monte Carlo; SFA: Total of saturated fatty acid; n-3: Total of omega 3; n-6: Total of omega 6.

## Competing interests

The authors declare that they have no competing interests.

## Authors’ contributions

ASMC, LCAR, LLC, RRT, GBM, DPDL, RTN and MMA conceived and designed the experiment; ASMC, LCAR, LLC, RTN, RRT, MLN, ASC and TSS performed the experiments; ASMC, LCAR, MAM, PSNO, DJG, JMR and LLC did analysis and interpretation of results; ASMC, LCAR, DJG, JMR and LLC drafted the manuscript. All authors read and approved the final manuscript.

## Supplementary Material

Additional file 1 **Manhattan plot of the genome-wide association study result for A) C12:0 (lauric acid) B) C16:0 (palmitic acid) C) C18:0 (stearic acid) in Nellore.** The X-axis represents the chromosomes, and the Y-axis shows the proportion of genetic variance explained by SNP window from Bayes B analysis. Click here for file

Additional file 2 Top 30 markers effect in BTA3 at 25 Mb associated with C18:0 and C18:1 cis-9 in Nellore steers. Click here for file

Additional file 3 **Manhattan plot of the genome-wide association study result for A) C18:3 n-3 (α-linolenic acid) B) C18:3 n-6 (α-linolenic acid) C) n-3 (total of n-3) in Nellore.** The X-axis represents the chromosomes, and the Y-axis shows the proportion of genetic variance explained by SNP window from Bayes B analysis. Click here for file

Additional file 4 Top three QTL regions associated with IMF deposition and composition traits in Nellore by Bayes B. Click here for file
