# Supplementary material for: Genome-wide association study for intramuscular fat deposition and composition in Nellore cattle
Source: BMC Genet. 2014 Mar 25;15:39. doi: 10.1186/1471-2156-15-39 (PMC4230646; doi:10.1186/1471-2156-15-39)
Supplement: Additional file 2 — Top 30 markers effect in BTA3 at 25 Mb associated with C18:0 and C18:1 cis-9 in Nellore steers. [file 1471-2156-15-39-S2.docx]

Additional file 4. Top 30 markers effect in BTA3 at 25 Mb associated with C18:0 and C18:1 cis-9 in Nellore steers.

| **BTA3 at 25 Mb** | | | |
| --- | --- | --- | --- |
| **SNP name** | **Marker Effect C18:0** | **Marker Effect 18:1 cis-9** | **Position**  **(Chr_bp)** |
| rs132903831 | 9.16E-03 | -9.64E-03 | 3_25871241 |
| rs109846051 | 7.74E-04 | -1.62E-02 | 3_25768532 |
| rs136685398 | 3.77E-04 | -8.07E-04 | 3_25856394 |
| rs133640984 | 3.10E-04 | -9.15E-04 | 3_25724431 |
| rs135852194 | 3.05E-04 | -4.65E-03 | 3_25924712 |
| rs135283257 | 1.94E-04 | -3.01E-03 | 3_25968578 |
| rs137571661 | 1.53E-04 | -4.49E-03 | 3_25935272 |
| rs110815636 | 1.31E-04 | -4.17E-04 | 3_25899926 |
| rs134138112 | 1.24E-04 | -1.10E-03 | 3_25822926 |
| rs133486019 | 1.07E-04 | -1.49E-04 | 3_25653435 |
| rs137726841 | 8.01E-05 | -2.06E-04 | 3_25434706 |
| rs43709927 | 7.45E-05 | -6.09E-05 | 3_25361462 |
| rs132978669 | 7.44E-05 | -4.05E-04 | 3_25683554 |
| rs134610317 | 6.80E-05 | -6.01E-05 | 3_25586625 |
| rs136526185 | 6.77E-05 | -1.45E-03 | 3_25971232 |
| rs132872603 | 6.60E-05 | -3.28E-04 | 3_25760484 |
| rs133573633 | 6.22E-05 | -2.19E-04 | 3_25750133 |
| rs110447077 | 5.05E-05 | -6.27E-05 | 3_25239334 |
| rs109380793 | 5.01E-05 | -7.29E-05 | 3_25125560 |
| rs133670803 | 4.94E-05 | -5.21E-05 | 3_25187122 |
| rs109125464 | 4.70E-05 | -1.88E-04 | 3_25654265 |
| rs133271406 | 4.51E-05 | -9.20E-05 | 3_25311952 |
| rs137417605 | 4.30E-05 | -5.14E-05 | 3_25332450 |
| rs134495781 | 4.23E-05 | -1.58E-04 | 3_25013585 |
| rs136479075 | 4.18E-05 | -1.39E-04 | 3_25720847 |
| rs109941469 | 4.17E-05 | -6.65E-05 | 3_25107210 |
| rs109791937 | 4.17E-05 | -1.37E-05 | 3_25451628 |
| rs136322611 | 4.04E-05 | -8.51E-05 | 3_25185439 |
| rs109561781 | 3.97E-05 | -4.94E-05 | 3_25593196 |
| rs134623220 | 3.81E-05 | -2.88E-05 | 3_25651862 |
