# Supplementary material for: Genome-wide association study for intramuscular fat deposition and composition in Nellore cattle
Source: BMC Genet. 2014 Mar 25;15:39. doi: 10.1186/1471-2156-15-39 (PMC4230646; doi:10.1186/1471-2156-15-39)
Supplement: Additional file 3 — Manhattan plot of the genome-wide association study result for A) C18:3 n-3 (α-linolenic acid) B) C18:3 n-6 (α-linolenic acid) C) n-3 (total of n-3) in Nellore. The X-axis represents the chromosomes, and the Y-axis shows the proportion of genetic variance explained by SNP window from Bayes B analysis. [file 1471-2156-15-39-S3.docx]

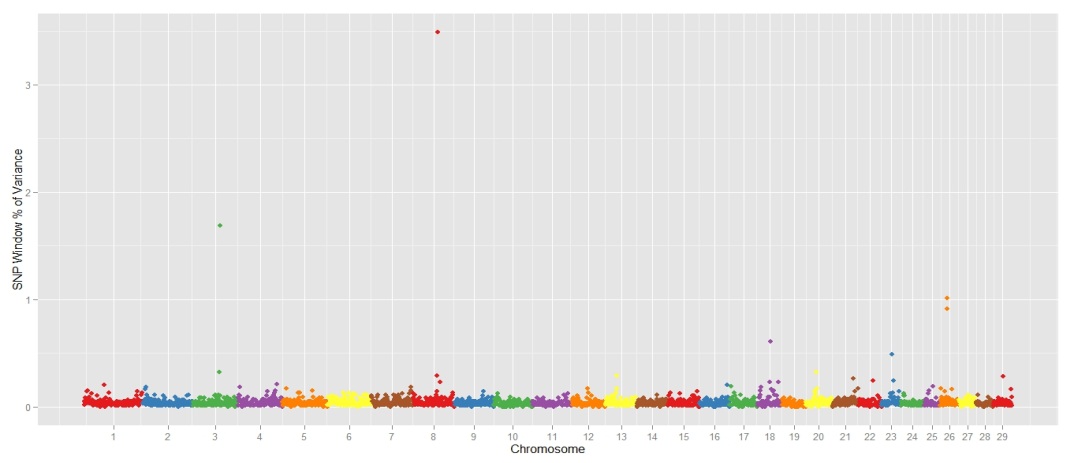

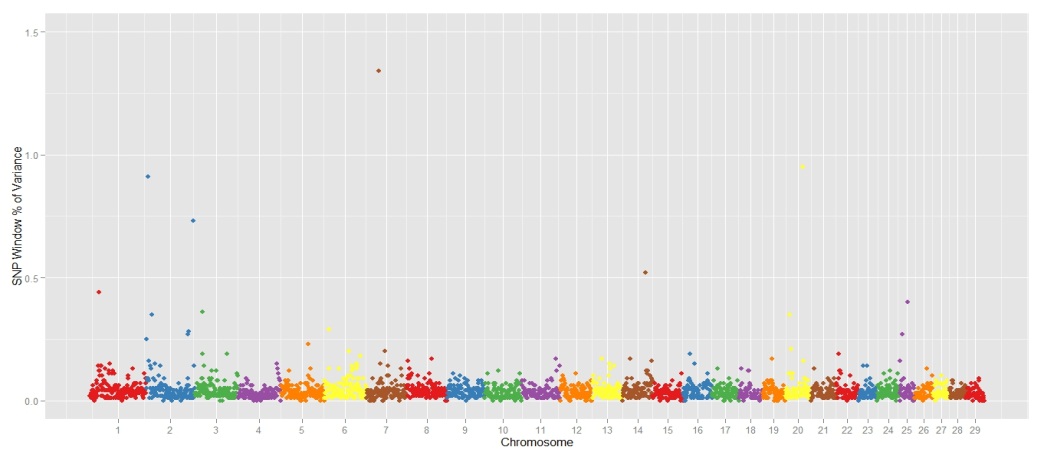

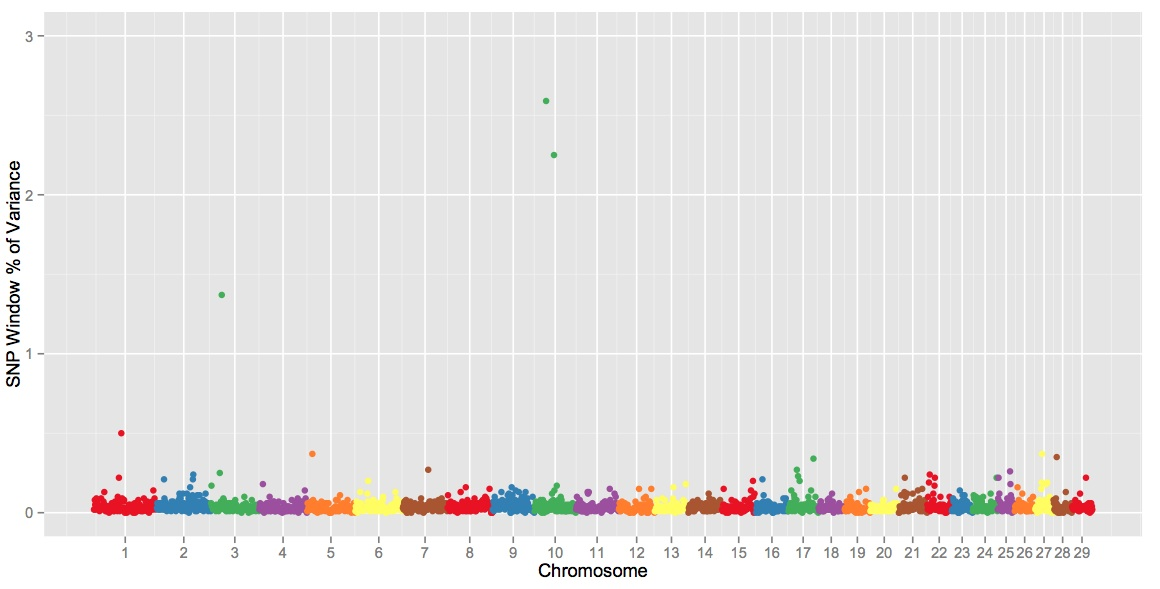


**B**

**C**

**A**

Additional file 3. Manhattan plot of the genome-wide association study result for A) C18:3 n-3 (γ-linolenic acid) B) C18:3 n-6 (α-linolenic acid) C) n-3 (total of n-3) in Nellore. The X-axis represents the chromosomes, and the Y-axis shows the proportion of genetic variance explained by SNP window from Bayes B analysis.
