# Supplementary material for: Genome-wide association study for intramuscular fat deposition and composition in Nellore cattle
Source: BMC Genet. 2014 Mar 25;15:39. doi: 10.1186/1471-2156-15-39 (PMC4230646; doi:10.1186/1471-2156-15-39)
Supplement: Additional file 4 — Top three QTL regions associated with IMF deposition and composition traits in Nellore by Bayes B. [file 1471-2156-15-39-S4.docx]

Additional file 1. Top three QTL regions associated with IMF deposition and composition traits in Nellore by Bayes B.

| **Trait** | **QTL Window**  **(first and last SNP)** | **Number of SNPs** | **%Gen.**  **Variance** | **Chr** | **Map Position**  **(UMD 3.1 bovine)** |
| --- | --- | --- | --- | --- | --- |
| IMF | rs110852801-rs110852801 | 144 | 0.66 | 10 | 50013104-50992412 |
|  | rs110396618-rs42932197 | 131 | 0.42 | 9 | 3018763-3997425 |
|  | rs109843584-rs137009265 | 243 | 0.4 | 6 | 28008364-28996379 |
| C12:0 | rs42924061-rs134942057 | 214 | 3.48 | 12 | 60000226-60994461 |
|  | rs135783220-rs109238095 | 169 | 0.44 | 5 | 38001237-38999839 |
|  | rs42639909-rs43332310 | 221 | 0.32 | 3 | 31001943-31992879 |
| C14:0 | rs41627556-rs110731616 | 332 | 1.43 | 9 | 36013595-36997263 |
|  | rs43328164-rs134696015 | 295 | 1.06 | 3 | 6009105-6999643 |
|  | rs134043558-rs134858042 | 182 | 0.82 | 2 | 107001640-107997035 |
| C14:1 cis-9 | rs137683417-rs29003360 | 232 | 1.86 | 2 | 26002605-26999718 |
|  | rs137754875-rs135670282 | 203 | 1.55 | 12 | 66002783-66997356 |
|  | rs110517663-rs137137362 | 127 | 1.43 | 11 | 27000446-27993515 |
| C15:0 | rs133645667-rs41591263 | 239 | 0.58 | 18 | 26001094-26997723 |
|  | rs133520595-rs135893631 | 199 | 0.45 | 14 | 75014305-75995520 |
|  | rs110569600-rs109846765 | 238 | 0.34 | 22 | 27024539-27994243 |
| C16:0 | rs109773631-rs135618512 | 277 | 1.53 | 12 | 13000697-13997298 |
|  | rs134160160-rs109942510 | 285 | 1.38 | 3 | 26002777-26998679 |
|  | rs135379047-rs136882159 | 173 | 0.58 | 8 | 20012045-20991197 |
| C16:1 cis-9 | rs133274959-rs136576856 | 213 | 1.42 | 3 | 25008982-25990012 |
|  | rs136405986-rs137105475 | 114 | 1.18 | 10 | 74001100-74957582 |
|  | rs135892505-rs109693564 | 184 | 1.04 | 7 | 80006438-80997126 |
| C17:0 | rs43471077-rs110039814 | 219 | 0.56 | 6 | 66005071-66997033 |
|  | rs137480735-rs137082211 | 153 | 0.51 | 4 | 38001142-38996557 |
|  | rs135375532-rs109902967 | 114 | 0.45 | 18 | 50001050-50870670 |
| C17:1 | rs110761991-rs110425294 | 83 | 0.83 | 5 | 55000572-55989100 |
|  | rs110775410-rs110218142 | 202 | 0.67 | 6 | 32002888-32998721 |
|  | rs133062684-rs109893445 | 144 | 0.6 | 6 | 21006361-21998823 |
| C18:0 | rs133274959-rs136576856 | 213 | 3.46 | 3 | 25008982-25990012 |
|  | rs132804279-rs109645596 | 102 | 2.08 | 11 | 105009792-105985714 |
|  | rs136405986-rs137105475 | 114 | 1.08 | 10 | 74001100-74957582 |
| C18:1 cis-9 | rs133274959-rs136576856 | 213 | 2.57 | 3 | 25008982-25990012 |
|  | rs109773631-rs135618512 | 277 | 1.91 | 12 | 13000697-13997298 |
|  | rs134160160-rs109942510 | 285 | 1.40 | 3 | 26002777-26998679 |
| C18:1 cis-11 | rs133850149-rs133020896 | 205 | 0.58 | 16 | 15004033-15997381 |
|  | rs135337759-rs43566804 | 192 | 0.51 | 8 | 76000301-76995458 |
|  | rs136472828-rs110306459 | 206 | 0.43 | 15 | 70000108-70998968 |
| C18:1 cis-12 | rs41776470-rs133872693 | 243 | 0.93 | 15 | 71015181-71997242 |
|  | rs137532692-rs134926660 | 186 | 0.44 | 24 | 49001908-49998425 |
|  | rs133496949-rs42611028 | 214 | 0.38 | 1 | 5004036-5976378 |
| C18:1 cis-15 | rs135695322-rs136588271 | 283 | 0.79 | 15 | 73014830-73997794 |
|  | rs135217991-rs133997168 | 260 | 0.48 | 11 | 13001510-13999856 |
|  | rs137648958-rs134302099 | 161 | 0.41 | 2 | 32007364-32996349 |
| C18:1 trans-6, 7, 8 | rs136989423-rs135647270 | 125 | 0.39 | 1 | 127000266-127991193 |
|  | rs134408743-rs134205414 | 117 | 0.37 | 9 | 25003343-25990361 |
|  | rs135780675-rs135123069 | 227 | 0.3 | 1 | 125001859-125996439 |
| C18:1trans-10, 11, 12 | rs136697703-rs137139554 | 122 | 0.57 | 5 | 90000699-90997254 |
|  | rs135750022-rs43138782 | 209 | 0.5 | 9 | 64000525-64992666 |
|  | rs137485825-rs133970673 | 110 | 0.49 | 13 | 58029906-58998257 |
| C18:1 trans-16 | rs133728493-rs109630757 | 264 | 2.56 | 6 | 50008629-50996673 |
|  | rs109872854-rs134277482 | 250 | 2.17 | 17 | 41001273-41991683 |
|  | rs109193786-rs109735608 | 215 | 0.62 | 2 | 65003697-65984693 |
| C18:2cis-9 cis-12 n-6 | rs134008882-rs136224281 | 164 | 0.46 | 9 | 52014336-52995340 |
|  | rs42780247-rs42794783 | 195 | 0.38 | 21 | 50005405-50997226 |
|  | rs43750576-rs42495887 | 212 | 0.31 | 12 | 62010947-62997021 |
| C18:2 cis-9 trans-11 | rs136630339-rs110298112 | 178 | 0.27 | 17 | 23004876-23997634 |
|  | rs134056892-rs377764129 | 222 | 0.26 | 8 | 27003611-27997814 |
|  | rs110518805-rs110823657 | 355 | 0.26 | 2 | 119002084-119974723 |
| C18:2 trans-11 cis-15 | rs135377389-rs133297940 | 267 | 3.49 | 8 | 68013821-68993778 |
|  | rs42404785-rs133183089 | 144 | 1.69 | 3 | 72000121-72997801 |
|  | rs137602675-rs133056879 | 187 | 1.01 | 26 | 20001875-20999270 |
| C18:3 n-3 | rs109612389-rs133661384 | 132 | 1.33 | 17 | 24002089-24987264 |
|  | rs41973816-rs109360228 | 142 | 0.43 | 21 | 21003963-21985795 |
|  | rs132831049-rs109343530 | 242 | 0.43 | 9 | 40014892-40995874 |
| C18:3 n-6 | rs42582725-rs133775322 | 205 | 1.34 | 7 | 34004443-34998434 |
|  | rs133846205-rs133219654 | 151 | 0.95 | 20 | 49025175-49991746 |
|  | rs136202159-rs134873098 | 235 | 0.91 | 2 | 5002853-5998856 |
| C20:1 | rs110940448-rs135112502 | 210 | 0.80 | 7 | 63000813-63990965 |
|  | rs134963634-rs42390829 | 207 | 0.79 | 20 | 55010178-55999450 |
|  | rs109443339-rs137113406 | 291 | 0.52 | 23 | 28001216-28998760 |
| C20:2 | rs136000962-rs136871508 | 213 | 0.26 | 1 | 63003623-63999110 |
|  | rs135902302-rs136049813 | 850 | 0.24 | 0 | unknown |
|  | rs42176463-rs133803377 | 319 | 0.20 | 29 | 35008674-35989729 |
| C20:3 n-6 | rs134302284-rs136338106 | 144 | 1.77 | 27 | 26000833-26975692 |
|  | rs136416983-rs134293611 | 373 | 0.38 | 1 | 69000931-69999565 |
|  | rs134477012-rs110532817 | 249 | 0.31 | 5 | 118004086-118995375 |
| C20:5 n-3 (EPA) | rs110411459-rs137802105 | 202 | 2.19 | 10 | 29000222-29962997 |
|  | rs137693004-rs135981990 | 198 | 0.66 | 12 | 1009795-1998903 |
|  | rs137823965-rs42507195 | 199 | 0.66 | 10 | 49005824-49980174 |
| C22:5 n-3 (DPA) | rs110411459-rs137802105 | 202 | 4.74 | 10 | 29000222-29962997 |
|  | rs132839318-rs134264692 | 306 | 3.35 | 3 | 27001604-27997941 |
|  | rs137823965-rs42507195 | 199 | 0.53 | 10 | 49005824-49980174 |
| C22:6 n-3 (DHA) | rs137823965-rs42507195 | 199 | 0.70 | 10 | 49005824-49980174 |
|  | rs109739360-rs137396104 | 232 | 0.46 | 7 | 99009734-99993114 |
|  | rs134412504-rs134289230 | 125 | 0.45 | 4 | 26004144-26999199 |
| MUFA | rs133274959-rs136576856 | 213 | 3.24 | 3 | 25008982-26998679 |
|  | rs134160160-rs109942510 | 285 | 1.13 | 3 | 26002777-26998679 |
|  | rs133803779-rs29003226 | 155 | 0.46 | 3 | 51006901-51976646 |
| PUFA | rs135174883-rs381709624 | 78 | 0.44 | 5 | 48109149-48993294 |
|  | rs109612389-rs133661384 | 132 | 0.42 | 17 | 24002089-24987264 |
|  | rs387618166-rs136897467 | 152 | 0.36 | 6 | 33002421-33992354 |
| n-3 | rs110411459-rs137802105 | 202 | 2.59 | 10 | 29000222-29962997 |
|  | rs137823965-rs42507195 | 199 | 2.25 | 10 | 49005824-49980174 |
|  | rs132839318-rs134264692 | 306 | 1.37 | 3 | 27001604-27997941 |
| n-6 | rs134302284-rs136338106 | 144 | 2.47 | 27 | 26000833-26975692 |
|  | rs132773171-rs42068328 | 123 | 0.77 | 25 | 33001144-33982544 |
|  | rs109016980-rs137260120 | 300 | 0.53 | 6 | 70000724-70995843 |
| PUFA:SFA | rs42522569-rs108981640 | 203 | 0.2 | 18 | 64004373-64999126 |
|  | rs137135250-rs136369955 | 257 | 0.19 | 14 | 28001989-28998719 |
|  | rs136562362-rs43211407 | 315 | 0.18 | 1 | 2009127-2995356 |
| n-6:n-3 | rs136309202-rs43468791 | 216 | 0.74 | 12 | 58000573-58990041 |
|  | BTA-38252-rs133950976 | 229 | 0.48 | 16 | 27024889-27996193 |
|  | rs135537573-rs133828431 | 275 | 0.43 | 22 | 57004878-57970902 |
| AI^1^ | rs135990954-rs134959509 | 283 | 0.19 | 1 | 68013613-68999267 |
|  | rs42964986-rs110471760 | 286 | 0.19 | 28 | 40001693-40996451 |
|  | rs135812645-rs110103414 | 297 | 0.18 | 9 | 102003073-102995933 |

^1^Atherogenic index = [12:0 + 4(14:0) + 16:0]/(SSFA + SPUFA).

^2^NS – Genomic region that explained less than 1% of genetic variance then was not studied.

^3^NG – Genomic region with no genes associated with the specific trait.
